# Phenotype-guided targeted therapy based on functional signal transduction pathway activity in recurrent ovarian cancer patients: The STAPOVER study protocol

**DOI:** 10.1016/j.heliyon.2023.e23170

**Published:** 2023-12-01

**Authors:** Phyllis van der Ploeg, Cynthia SE. Hendrikse, Anna MJ. Thijs, Hans M. Westgeest, Huberdina PM. Smedts, M Caroline Vos, Mathilde Jalving, Christianne AR. Lok, Ingrid A. Boere, Maaike APC. van Ham, Petronella B. Ottevanger, Anneke M. Westermann, Constantijne H. Mom, Roy I. Lalisang, Sandrina Lambrechts, Ruud LM. Bekkers, Jurgen MJ. Piek

**Affiliations:** a Department of Obstetrics and Gynaecology and Catharina Cancer Institute, Catharina Hospital, Eindhoven, the Netherlands; b GROW School for Oncology and Reproduction, Maastricht University, Maastricht, the Netherlands; c Department of Internal Medicine and Catharina Cancer Institute, Catharina Hospital, Eindhoven, the Netherlands; d Department of Internal Medicine, Amphia Hospital, Breda, the Netherlands; e Department of Obstetrics and Gynaecology, Amphia Hospital, Breda, the Netherlands; f Department of Obstetrics and Gynaecology, Elisabeth-Tweesteden Hospital, Tilburg, the Netherlands; g Department of Medical Oncology, University Medical Centre Groningen, Groningen, the Netherlands; h Department of Gynaecologic Oncology, Netherlands Cancer Institute-Antoni van Leeuwenhoek Hospital, Amsterdam, the Netherlands; i Department of Medical Oncology, Erasmus Medical Centre Cancer Institute, Rotterdam, the Netherlands; j Department of Obstetrics and Gynaecology, Radboud University Medical Centre, Nijmegen, the Netherlands; k Department of Oncology, Radboud University Medical Centre, Nijmegen, the Netherlands; l Department of Oncology, Amsterdam University Medical Centre, Amsterdam, the Netherlands; m Department of Obstetrics and Gynaecology, Amsterdam University Medical Centre, Amsterdam, the Netherlands; n Department of Medical Oncology, Maastricht University Medical Centre +, Maastricht, the Netherlands; o Department of Obstetrics and Gynaecology, Maastricht University Medical Centre +, Maastricht, the Netherlands

**Keywords:** Ovarian cancer, Signal transduction pathways, Survival, targeted therapy, Off-label drugs

## Abstract

**Objective:**

Ovarian cancer is the fifth cause of cancer-related death among women. The benefit of targeted therapy for ovarian cancer patients is limited even if treatment is stratified by molecular signature. There remains a high unmet need for alternative diagnostics that better predict targeted therapy, as current diagnostics are generally inaccurate predictors. Quantitative assessment of functional signal transduction pathway (STP) activity from mRNA measurements of target genes is an alternative approach. Therefore, we aim to identify aberrantly activated STPs in tumour tissue of patients with recurrent ovarian cancer and start *phenotype*-guided targeted therapy to improve survival without compromising quality of life.

**Study design:**

Patients with recurrent ovarian cancer and either 1) have platinum-resistant disease, 2) refrain from standard therapy or 3) are asymptomatic and not yet eligible for standard therapy will be included in this multi-centre prospective cohort study with multiple stepwise executed treatment arms. Targeted therapy will be available for patients with aberrantly high functional activity of the oestrogen receptor, androgen receptor, phosphoinositide 3-kinase or Hedgehog STP. The primary endpoint of this study is the progression-free survival (PFS) ratio (PFS2/PFS1 ratio) according to RECIST 1.1 determined by the PFS on matched targeted therapy (PFS2) compared to PFS on prior therapy (PFS1). Secondary endpoints include among others best overall response, overall survival, side effects, health-related quality of life and cost-effectiveness.

**Conclusion:**

The results of this study will show the clinical applicability of STP activity in selecting recurrent ovarian cancer patients for effective therapies.

**Trial registration number**: NL2020-005091-36 (EudraCT) and NCT03458221 (ClinicalTrials.gov).

## 1 Introduction

Ovarian cancer is the fifth leading cause of cancer death among women in the Western world [1]. The majority of the patients is diagnosed with advanced stage disease, which requires extensive cytoreductive surgery in combination with platinum and paclitaxel containing chemotherapy [2]. Despite this extensive treatment, recurrent disease almost invariably occurs resulting in a five-year survival rate ranging between 20% and 41% [3], which has not changed significantly over the last decades [4]. Recurrent ovarian cancer could be treated with second-/third-line chemotherapy and targeted therapies, such as poly(ADP-ribose) polymerase (PARP) inhibitors [2]. Although other targeted therapy options are rapidly expanding [5], response rates often fall short of expectations [6]. This can be the result of suboptimal selection based on inadequate molecular diagnostics. Therefore, there is an urgent need to improve patient stratification methods to allocate patients with ovarian cancer to effective targeted therapies and prevent unnecessary side effects of ineffective therapies.

Tumour cell proliferation, differentiation and migration is often driven by aberrant cellular activity of signal transduction pathways (STPs) [7]. STPs can be categorized as nuclear receptor pathways (e.g. oestrogen receptor (ER) and androgen receptor (AR)), developmental pathways (e.g. Hedgehog (HH), transforming growth factor beta (TGF-β), Notch), and highly complex growth factor regulated pathways (e.g. phosphoinositide 3-kinase (PI3K) and mitogen-activated protein kinase (MAPK)). During the past decades, our knowledge on the complex mechanisms of action of these STPs has expanded and studies demonstrated associations between aberrant STP activity and ovarian carcinogenesis [[8], [9], [10], [11], [12], [13], [14]].

The introduction of whole genome sequencing technologies enabled the identification of genomic alterations associated with these STPs in tumour samples. Unfortunately, the implementation of genomic-based targeted therapy has not been as successful as initially expected [15]. Although a proportion of patients in molecular profiling studies demonstrates clinical benefit, the majority of these carefully selected patients lack response to costly targeted drugs [16,17]. These findings suggest that we have overestimated the contribution of the cancers’ *genotype* in predicting therapy response, but it could be questioned if the sole presence of gene alterations (e.g. gene mutations or amplifications) is sufficient to provide information on the functional activation status of STPs. Until now, the *functional phenotype* of tumour cells that is influenced by other factors, such as the tumour microenvironment is often disregarded.

In recognition of the significance of the cancer's *phenotype*, an alternative approach to assess STP activity was developed using Bayesian computational network models [[18], [19], [20], [21], [22], [23]]. The models infer STP activity from mRNA measurements of well-validated direct target genes of the transcription factor complex associated with the respective STP from paraffin-embedded tumour samples. Until now, models have been developed for the ER, AR, HH, TGF-β, Notch, PI3K, and MAPK pathways. While current approaches focus on a single molecular trait, these models use expression levels of several pathway-specific target genes and are therefore thought to be a more precise way to interpret functional STP activity. Previous studies including other tumour types, such as breast and salivary duct cancer, suggested the mRNA-based assay to be of value in the selection of patients for targeted therapy based on activated STPs [[24], [25], [26]].

Our research group investigated STP activity in several subtypes of ovarian cancer to identify which STPs could be potential targets for personalized medicine [27,28]. Furthermore, we determined STP activity in morphologically normal Fallopian tube epithelium (FTE) of healthy women, the considered tissue of origin of most high-grade serous carcinoma (HGSC), to establish normal STP activity as a reference. [29] In the current study, we aim to determine actionable tumour-promoting STPs in tumour samples of recurrent ovarian cancer patients based on these results and implement *phenotype*-guided targeted therapy to improve survival without compromising quality of life. Therefore, we propose a prospective, parallel-group cohort study involving patients with recurrent ovarian cancer to be treated with approved targeted drugs, if identified with an aberrantly activated STP.

## 2 Methods

### 2.1 Study design

**Fig. 1:**
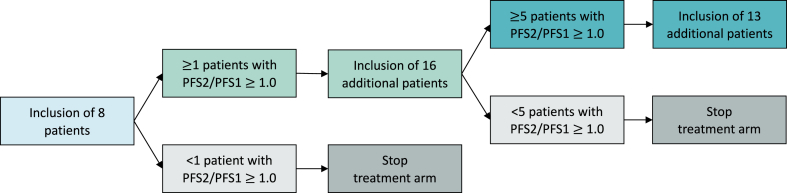
Flowchart displaying the three-stage design for inclusion of patients per treatment arm. PFS; progression-free survival.

The Signal TrAnsduction Pathway activity analysis for OVarian cancER treatment (STAPOVER) study is an investigator-initiated, multi-centre, prospective, parallel-group cohort study with stepwise executed treatment arms. Patients with platinum-resistant recurrent ovarian cancer, patients with recurrent ovarian cancer who refrain from standard therapy and patients with recurrent ovarian cancer who are not yet eligible for standard palliative chemotherapy due to asymptomatic disease, will be included to receive targeted therapy based on aberrantly active STPs, identified by the STP activity assay. Patients diagnosed with any histological subtype of ovarian cancer are eligible for inclusion in the study. The rationale for including all histological subtypes is driven by the need to move beyond histological classifications and focus on molecular profiling. In the individual treatment arms, a modified version (three-stage) of Simon's two-stage design is incorporated (Fig. 1) [30].

### 2.2 Eligibility criteria

Adult women (age ≥18 years) diagnosed with recurrent ovarian cancer of any histological subtype who either 1) have platinum-resistant disease, defined as disease recurrence or progression within six months of last platinum-based chemotherapy (e.g. cisplatin or carboplatin) 2) refrain from standard therapy or 3) are asymptomatic and not yet eligible for standard therapy with an increased CA125 tumour marker of a nadir above 35 U/ml for at least 28 days, are eligible for enrolment when the following inclusion criteria are met.

• Progressive disease after at least one prior line of systemic treatment for recurrent disease.
• Radiologically evaluable disease according to RECIST 1.1 [31].
• Ability and willingness to provide a tumour biopsy after the last course of standard treatment and before start of the study.
• Ability and willingness to provide written and oral consent.
• Able to speak and understand the Dutch language.
• WHO performance status 0-II.
• Adequate renal and liver function to start matched targeted therapy (according to the local clinician).
• Adequate use of contraceptives in case of patients with childbearing potential.

Women are not eligible to participate in the study in case any of the following exclusion criteria are met.

• Patient is receiving any other anti-cancer therapy or is chemotherapy naïve. The required wash-out period prior to the start of matched targeted therapy is at least three weeks.
• Patient is diagnosed with or treated for a second primary tumour (except non-melanoma skin tumour) one year prior to study inclusion.
• Inability to obtain (sufficient) tumour material.
• Previous use of the selected targeted drug as anti-cancer agent.
• Pregnant or lactating women.
• Simultaneous participation in another treatment-related clinical trial.
• Patients with any other clinically significant medical condition which, in the opinion of the local clinician, makes it undesirable for the patient to participate in this study or which could jeopardize compliance with study requirements.

### 2.3 Study procedures

**Fig. 2:**
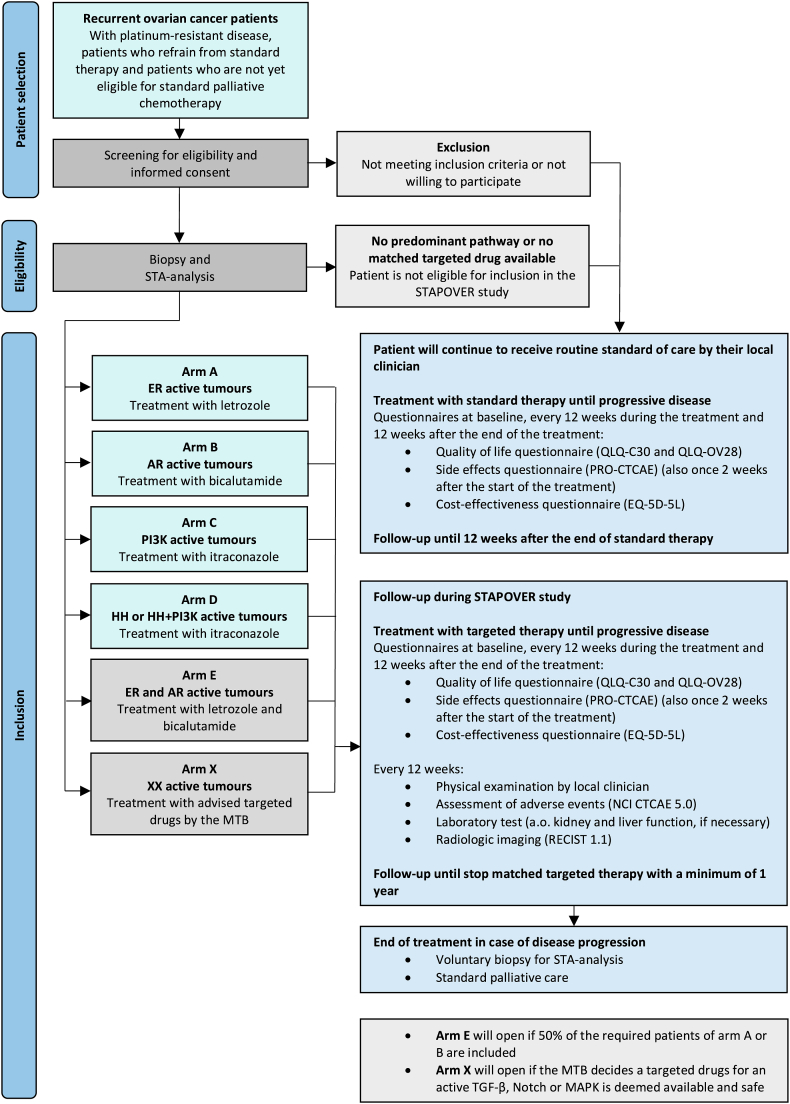
Flowchart of the study design. STP; signal transduction pathway, ER; oestrogen receptor, AR; androgen receptor, HH; Hedgehog, PI3K; phosphoinositide 3-kinase, TGF-β; transforming growth factor beta, MAPK; mitogen activated protein kinase.

Informed consent will be obtained from the patients who are willing to participate and meeting the inclusion criteria. Subsequently, one histological biopsy will be collected to obtain tumour tissue for the STP activity assay. Patients are eligible for treatment with matched targeted therapy if an aberrantly activated STP is observed. Women with high activity of the ER, AR, PI3K or HH pathway will be included for treatment with either letrozole (arm A), bicalutamide (arm B), or itraconazole (arm C and D) (Fig. 2). These targeted drugs have been studied in ovarian cancer patients with acceptable side effects [[32], [33], [34], [35], [36], [37], [38], [39], [40], [41], [42]]. Patients who are not eligible to participate in the study will continue to receive standard of care by choice of the treating clinician. These patients are asked for consent to remain on study to complete questionnaires at baseline, every 12 weeks during the next course of treatment and 12 weeks after the end of treatment. Questionnaires are used for secondary analyses, such as side effects, quality of life and cost-effectiveness.

### 2.4 Tumour sampling

Tumour tissue will be obtained from at least one tumour site through a biopsy guided by ultrasound or CT-scan, or through a (pre-planned) cytoreductive surgical procedure. In case of multiple tumour sites, a tumour site based on accessibility and safety risks will be selected. Successfully obtained tumour tissue will be formalin-fixed paraffin-embedded (FFPE) by the hospital's histopathological laboratory. In case biopsy fails, e.g. because of insufficient tumour tissue or no accessible tumour site, the patient will be excluded from the study.

### 2.5 Preparation of tumour samples

In addition to standard diagnostics, for the STAPOVER study at least 20 sections of 5 μ m will be cut from the FFPE sample with a microtome. The first and last study slide will be haematoxylin and eosin (H&E) stained. The H&E-stained slide will be revised by a local pathologist to annotate a tumour area containing the highest amount of tumour cell nuclei (≥50 %).

The coordinating centre will provide a study number for each individual patient to label the study slides before they will be transported to InnoSIGN for STP activity assay. The remainder of the FFPE samples and slides for standard diagnostics will be archived in the participating centre. If sufficient study slides have been obtained, several slides will be archived by the coordinating centre for optional analysis (such as immunohistochemical biomarker analysis and DNA/RNA sequencing).

### 2.6 STP activity assay and determination of high STP activity

Using the annotated H&E slide as reference, tumour cells will be manually scraped off the study slides for the STP activity assay. Next, mRNA will be isolated from the tumour cells using the RNeasy FFPE Kit (Qiagen). Expression levels of pathway-specific target genes will be measured with real-time quantitative reverse transcription-PCR (RT-qPCR) analysis using the SuperScript III Platinum One-Step qRT-PCR Kit (Thermo Fisher Scientific) and commercially available OncoSIGNal 96-wells PCR plates (InnoSIGN). An internal quality control of reference genes is performed to confirm sufficient mRNA input for the assay.

**Fig. 3:**
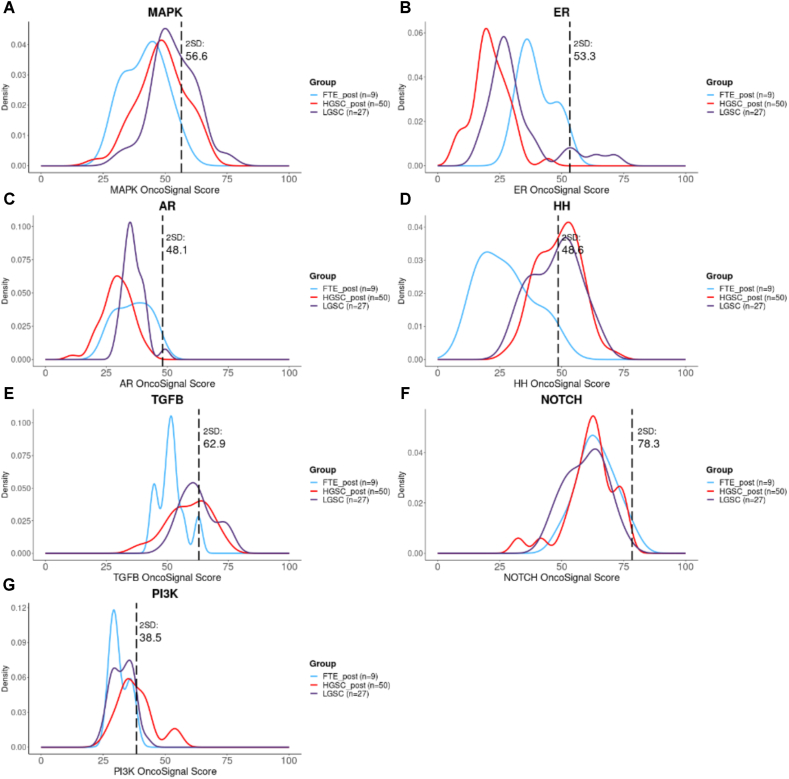
Example of the standard format for signal transduction pathway (STP) activity assay data presentation to the multidisciplinary tumour board. The figure shows curves of the STP activity of a cohort high-grade serous ovarian carcinoma (HGSC, n = 50) in red, a cohort of low-grade serous ovarian carcinoma (LGSC, n = 27) in purple and a cohort of Fallopian tube epithelium samples of healthy postmenopausal women (FTE_post, n = 9) in blue, for each of the seven measured STPs. The STP specific thresholds are defined as two standard deviations above the mean value of the STP activity measured in the Fallopian tube epithelium samples (n = 9). A. MAPK; mitogen activated protein kinase, B. ER; oestrogen receptor, C. AR; androgen receptor, D. HH; Hedgehog, E. TGFB; transforming growth factor beta, F. NOTCH, G. PI3K; phosphoinositide 3-kinase. (For interpretation of the references to colour in this figure legend, the reader is referred to the Web version of this article.)

STP activity assay provides a functional pathway activity score defined on a scale from 0 to 100, where the measured range of pathway activity may vary with the analysed tissue type. A score of 0 corresponds with the lowest odds of an active pathway and conversely 100 corresponds with the highest odds. In a prior study, we quantified the activity of STPs in epithelial cells derived from morphologically normal Fallopian tubes[29]. This analysis served as a basis to establish the reference levels for normal STP activity. We defined cut-off values for high STP activity in tumour samples, which were determined as two standard deviations above the mean value of STP activity measured in FTE samples. Individual STP activity assay results will be presented and analysed in a graph as shown in Fig. 3. This format contains the STP activity distributions measured in previous cohorts of normal FTE (blue curve), HGSC samples (red curve) and low-grade serous ovarian carcinoma (LGSC) samples (purple curve) [28,29,43]. For each measured STP, data is displayed in a single graph. The black dotted line represents the cut-off value for high STP activity. Before the start of the study, cut-off values will be updated to values in which the most recent results of the STP activity of the normal FTE are included. The individual STP activity measured in the patient's tumour sample will be plotted in the format. In case the individual STP activity transcends the cut-off (black dotted line), the STP will be classified as aberrantly activated. STP activity results will be sent to the principal investigators, who share the results with the multidisciplinary tumour board (MTB).

### 2.7 Multidisciplinary tumour board

The nationwide MTB will consist of gynaecological oncologists, medical oncologists and a translational researcher (trial manager). The MTB will decide on matched targeted therapy based on the STP activity assay results, their scientific experience and the available literature. STP activity results will be presented to the MTB in a standard format (Fig. 3). An MTB selection form has been developed to assist in guiding the selection of matched targeted therapy based on aberrantly activated STPs. In scenarios where multiple STPs show abnormal activation, a predefined set of questions within the selection form aids in decision-making. The form includes an assessment of which pathway exceeds the defined threshold value to the greatest extent, enabling prioritized inhibition of that particular pathway using targeted therapy. If the MTB decides the patient is eligible for matched targeted therapy, treatment will be initiated by the local clinician within 28 days. In case no aberrantly activated pathway is identified, the patient will receive standard of care.

### 2.8 Interventions

Women diagnosed with tumours with aberrantly high activity of the ER, AR, PI3K or HH STP, will receive the following treatment with the recommended dosage (Fig. 2).

• Treatment arm A (ER active tumours) with aromatase-inhibitor letrozole 2.5 mg daily.
• Treatment arm B (AR active tumours) with anti-androgen bicalutamide 150 mg daily.
• Treatment arm C (PI3K active tumours) with anti-fungal agent itraconazole 300 mg twice daily.
• Treatment arm D (HH or concurrent HH and PI3K active tumours) with anti-fungal agent itraconazole 300 mg twice daily.

The selection of these targeted drugs was based on specific criteria. These criteria included demonstrated effectiveness in a subset of ovarian cancer patients or evidence of inhibition of pathway activity in cancer samples. Furthermore, all selected drugs were required to have received approval from the European Medicines Agency (EMA) for clinical use and be commercially available in the Netherlands. The drugs will be used outside their therapeutic indications during this study. A combination of two drugs targeting the ER and AR pathway is allowed if advised by the MTB (arm E) (Fig. 2).

Other STPs (TGF-β, Notch and MAPK) may be targeted during the study period by other available drugs, provided there is sufficient evidence for its use and safety. If deemed beneficial, the MTB could decide to open a new treatment arm (arm X) (Fig. 2).

### 2.9 Pre-treatment evaluation before start of matched targeted therapy

Before the start of the recommended matched targeted therapy, patients will undergo a pre-treatment evaluation by their treating clinician. Baseline characteristics will be registered in the electronic case report form (eCRF). Furthermore, physical examination will be performed and the treating clinician will determine if any safety concerns exist. The treating clinician will assess if the patient has adequate renal and kidney function and checks for interactions between the matched targeted therapy and concomitant medication. Imaging of the tumour and evaluation by RECIST 1.1 of the tumour will be performed at baseline [31].

### 2.10 Follow-up during matched targeted therapy

The treating clinician will be responsible for adequate follow-up during treatment with matched targeted therapy. Response will be evaluated at least every 12 weeks by imaging of the tumour according to RECIST 1.1 [31]. Additionally, physical examination and assessment of liver and kidney function are performed. Adverse events are evaluated according to the NCI CTCAE 5.0.

Both patients using matched targeted therapy, as well as patients who were not eligible to participate in the study and continued to receive standard of care, will be asked to complete the questionnaires to investigate health-related quality of life (QLQ-C30 and QLQ-OV28) [44], side effects (PRO-CTCAE) and cost-effectiveness (EQ-5D-5L) [45] at baseline, every 12 weeks during treatment and 12 weeks after the end of the treatment.

### 2.11 End of treatment

In case of disease progression, patients will be withdrawn from the study and treatment with the matched targeted therapy will be ended. After disease progression, it is not possible to be included in the study for a second time; patients will receive standard (palliative) care. Patients will be asked to undergo a voluntary second biopsy to determine STP activity for research purposes after treatment has ended and before other systemic treatment has started.

## 3 Results

### 3.1 Outcome measures

The primary objective of this study is the progression-free survival (PFS) ratio (PFS2/PFS1 ratio) defined by the PFS on matched targeted therapy by STP activity assay (PFS2) in comparison to the PFS recorded on the therapy administered immediately prior to enrolment (PFS1) [31].

Secondary outcome measures include.

• Proportion of patients with an actionable active STP for which targeted therapy is recommended in relation to the number of patients who underwent a biopsy.
• Proportion of patients who receive matched targeted therapy in relation to the number of patients included in each study arm.
• Best overall response defined by RECIST 1.1 based on radiological imaging [31].
• One-year survival defined as the time from start matched targeted therapy till death or the end of the one-year follow-up period.
• Overall survival defined as the time from start matched targeted therapy till death.
• Predictive value of STP activity assay results (and if available, immunohistochemical biomarker analysis and DNA/RNA sequencing results) on matched targeted therapy response.
• Change in STP activity score after disease progression (if available) compared to STP activity score before start of matched targeted therapy.
• Side effects according to PRO-CTCAE™.
• Health-related quality of life scored using standardized questionnaires from the EORTC (QLQ-C30 and QLQ-OV28) [44].
• Cost-effectiveness will be calculated with health state utilities assessed in the standardized EuroQol 5D (EQ-5D-5L) questionnaire [45].

### 3.2 Sample size

In this study, the PFS2/PFS1 ratio will be used as primary outcome measure, in which the correlation of two consecutive lines of treatment allows the patient to serve as her own control and compensates for heterogeneity in patient characteristics and tumour histology. Two prior prospective studies used the PFS2/PFS1 ratio to measure treatment response on targeted therapies based on molecular profiling in patients with advanced cancers [17,46]. Both studies deemed a PFS2/PFS1 ratio ≥ 1.3 to clinically beneficial. Their null hypothesis stated that ≤ 15 % of the patient population would have a PFS2/PFS1 ratio of 1.3 on standard therapy. In order to reject the null hypothesis, the authors assumed that the true proportion of patients with a PFS2/PFS1 ratio ≥ 1.3 in the studies would be equal to 30 % [46] and 24 % [17].

Based on these results, we calculated that 37 evaluable patients are required per treatment arm (total of 148 patients for treatment arm A, B, C and D) to reject the null hypothesis of an estimated 15 % of patients achieving PFS2/PFS1 ratio ≥1.3 on standard palliative therapy with 80 % power at a one-sided significance level of 0.10.

Per stepwise executed treatment arm, interim analyses will be performed after the inclusion of the first eight and 24 patients (Fig. 1) [30]. In case none of the first eight patients achieve PFS2/PFS1 ≥ 1.0, the treatment arm will be closed due to lack of efficacy. The treatment arm will be complemented by 16 additional patients if at least one of the first eight patients achieves PFS2/PFS1 ≥ 1.0. In case at least five of the enrolled 24 patients achieve PFS2/PFS1 ≥ 1.0, the treatment arm is considered a potentially successful treatment and may proceed with enrolment until 37 patients have been included. If four or less patients achieve PFS2/PFS1 ≥ 1.0, the treatment arm will be closed due to insufficient efficacy. For this monitoring rule, we choose the threshold of 1.0 to represent sufficient patient benefit to continue patient accrual. After closing a treatment arm, it is allowed to open a new treatment arm targeting the same STP with a new targeted drug, if this is deemed beneficial by the MTB.

Patients who withdraw from the study before start of matched targeted therapy will be replaced until a total of 37 patients are available for response measurements. If a patient withdraws from the study after the start of matched targeted therapy, the patient will not be replaced and will be included in the analysis.

Our research group investigated STP activity in previous cohorts of women with HGSC and LGSC [28,29,43]. Based on our observations in these studies, we anticipate the presence of an active ER, AR, PI3K or HH pathway in approximately 50 % of the patients included in the study. It is expected that approximately 10 % of the cases will result in biopsies with insufficient tumour percentages. Additionally, considering a drop-out rate of 10 % prior to the initiation of targeted therapy, we estimate that a total of 366 patients will need to undergo biopsy to achieve intended sample size for this study. We estimate to be able to recruit sufficient number of patients for study arm A, B, C and D in five years, in a total of six participating centres in the Netherlands.

### 3.3 Plan of analysis

The proportion of patients who achieved a PFS2/PFS1 ratio ≥ 1.3 will be calculated from the total of patients that received matched targeted therapy. The primary endpoint will be tested by a one-sided exact test. Since histological subtype is a prognostic factor for PFS [36], subgroup analysis will be performed in case of adequate numbers.

Planned statistical analysis for the secondary outcome measures include the Kaplan-Meier survival curves tested with log-rank tests and Cox proportional hazard models for survival analysis. Furthermore, the predictive value of STP activity assay will be determined by correlations between STP activity scores and PFS2 using the Pearson or Spearman correlation coefficient. If patients undergo a voluntary second post-treatment biopsy, change in STP activity score will be calculated for each respective STP compared to the STP scores from the pre-treatment biopsy.

Using the appropriate algorithms, the side effects and health-related quality of life questionnaires will be analysed and compared accordingly between the group of patients that received matched targeted therapy and patients that received standard therapy. Together with the PFS, these results will be used in a quality adjusted life year calculation in order to calculate the cost-effectiveness and cost-utility ratios of matched targeted therapy. In addition, subgroup analysis per treatment arm will be conducted.

Descriptive statistics will be used to describe patient characteristics. Categorical variables will be expressed as a number with the percentage of the total study group. The chi-squared test or Fisher's exact test will be used to analyse associations between categorical variables. Continuous variables will be presented as mean with standard deviation or as median with interquartile range, whenever appropriate. A *t*-test or Mann-Whitney *U* test will be used to analyse associations between continuous variables.

### 3.4 Ethics and dissemination

The study will be conducted according to the principles of the Declaration of Helsinki (amended by the 68th WMA General Assembly, Chicago, United States, October 2017). All researchers involved in the study will follow the Dutch Medical Research Involving Human Subjects Act and ‘Code Goed Gebruik’ (Federa 2002, amended in 2011). Ethical approval for the study has been obtained from the Medical research Ethics Committees United (MEC-U, study number R21.033) and Central Committee on Human Research (CCMO, study number NL77022.100.21). The study has been registered with the European Union Drug Regulating Authorities Clinical Trials (EudraCT) database under reference number NL2020-005091-36 and with ClinicalTrials.gov under reference number NCT03458221.

All participants will be informed on the purpose, procedure, duration and possible risks and benefits of the study. Participants need to provide written informed consent prior to study inclusion and, in case a matched targeted drug is available and recommended, prior to the start of therapy. Participants can withdraw consent to participate at any time for any reason and without any consequences.

All data will be handled strictly confidential and will be collected using an eCRF in the secured database Research Manager (https://my-researchmanager.com). Data and tumour tissues will be coded with a random study number and will be stored for at least 15 years after the end of the study.

Study results of individual cohorts or pooled data will be offered to international peer-reviewed journals for publication and presented at conferences. Publication will not disclose the identity of the participants.

## 4 Conclusion

This is the first study to evaluate the clinical implementation of *phenotype*-guided targeted therapy based on STP activity in tumour tissue of patients with recurrent ovarian cancer. Patients are treated with existing targeted drugs with tolerable toxicity profiles to investigate whether these drugs have therapeutic value beyond their approved indications. The unique study design allows for simultaneous screening for several promising treatment strategies and rapid incorporation of new cohorts based on other (off-label) targeted drugs or STPs. Intra-individual comparison of treatment response through the PFS ratio will limit confounders such as tumour histology. The results of this study will show the clinical applicability of STP activity to match recurrent ovarian cancer patients to targeted therapies.

## Funding

This work is supported by the Catharina Research fund [grant number 2022–3] and InnoSIGN (previously Molecular Pathway Diagnostics, 10.13039/100004320Philips). Furthermore, this work is co-financed by the European Union through the subsidy program OPZUID (project number STIM-00107).

## CRediT authorship contribution statement

**Phyllis van der Ploeg:** Conceptualization, Methodology, Writing – original draft. **Cynthia SE. Hendrikse:** Conceptualization, Methodology, Writing – original draft. **Anna MJ. Thijs:** Conceptualization, Methodology, Writing – review & editing. **Hans M. Westgeest:** Conceptualization, Methodology, Writing – review & editing. **Huberdina PM. Smedts:** Conceptualization, Methodology, Writing – review & editing. **M Caroline Vos:** Conceptualization, Writing – review & editing. **Mathilde Jalving:** Conceptualization, Writing – review & editing. **Christianne AR. Lok:** Conceptualization, Writing – review & editing. **Ingrid A. Boere:** Conceptualization, Writing – review & editing. **Maaike APC. van Ham:** Conceptualization, Writing – review & editing. **Petronella B. Ottevanger:** Conceptualization, Writing – review & editing. **Anneke M. Westermann:** Conceptualization, Writing – review & editing. **Constantijne H. Mom:** Conceptualization, Writing – review & editing. **Roy I. Lalisang:** Conceptualization, Writing – review & editing. **Sandrina Lambrechts:** Conceptualization, Methodology, Supervision, Writing – review & editing. **Ruud LM. Bekkers:** Conceptualization, Methodology, Supervision, Writing – review & editing. **Jurgen MJ. Piek:** Conceptualization, Methodology, Supervision, Writing – review & editing.

## Declaration of competing interest

The authors declare the following financial interests/personal relationships which may be considered as potential competing interests: Jurgen Piek reports equipment, drugs, or supplies was provided by InnoSIGN. Phyllis van der Ploeg reports a relationship with Catharina Research Fund that includes: funding grants. Cynthia Hendrikse reports a relationship with Catharina Research Fund that includes: funding grants. Phyllis van der Ploeg reports a relationship with InnoSIGN that includes: funding grants. Cynthia Hendrikse reports a relationship with InnoSIGN that includes: funding grants.
