# Supplementary material for: Phenotype-guided targeted therapy based on functional signal transduction pathway activity in recurrent ovarian cancer patients: The STAPOVER study protocol
Source: Heliyon. 2023 Dec 1;10(1):e23170. doi: 10.1016/j.heliyon.2023.e23170 (PMC10770441; doi:10.1016/j.heliyon.2023.e23170)
Supplement: Multimedia component 1 [file mmc1.pdf]

Datum:

		-			-					

Studienummer:

STAPOVER studie

Signaaltransductie pad activiteit analyse voor de behandeling van eierstokkanker

Instructies en toelichting op de vragenlijst
Deze vragenlijst maakt deel uit van de studie naar het effect van behandeling met doelgerichte therapie op basis van signaaltransductie pad activiteit in vrouwen met eierstokkanker. Wij willen u vragen de instructies rustig door te lezen. Vult u alstublieft alle vragen in, ook wanneer deze voor u minder van belang zijn. Hoewel sommige vragen op elkaar kunnen lijken, is toch iedere vraag weer anders. Als u twijfelt, geef dan het antwoord dat het dichtst in de buurt komt van uw situatie. U kunt steeds antwoord geven door het hokje aan te kruisen dat het beste op u van toepassing is. Bij sommige vragen kunt u getallen invullen in de aangewezen vakjes. Er zijn geen goede of foute antwoorden; het gaat uitsluitend om uw persoonlijke mening. Alle antwoorden zullen anoniem worden verwerkt en zijn daardoor nooit tot u herleidbaar. Er wordt geen contact met u opgenomen naar aanleiding van de antwoorden die u heeft ingevuld op deze vragenlijst.

Mocht u nog vragen of opmerkingen hebben naar aanleiding van deze vragenlijst, dan kunt u contact opnemen met de studiecoördinatoren.

Alvast hartelijk dank voor uw medewerking.

Studiecoördinatoren: dr. Jurgen Piek en dr. Annemarie Thijs
Adres: Catharina Ziekenhuis, Michelangelolaan 2, 5623 EJ Eindhoven
Telefoonnummer: 040 - 239 93 00
E-mailadres: stapover@catharinaziekenhuis.nl

Invulinstructies

- Vul de vragenlijst in met een zwarte of blauwe pen (geen viltstift).
- Zet een duidelijk kruisje in het antwoordvakje.
- Als u een fout antwoord invult, laat dan het foutieve kruisje staan en maak het goede vakje helemaal zwart.
- Vul bij een getal één cijfer per vakje in. Het hele cijfer moet binnen het vakje komen. Geen streepjes zetten als u iets niet hoeft in te vullen.
- Kruis bij elke vraag één hokje aan.

Algemene vragen

1. Uw geboortejaar: ☐☐☐☐

2. Wat is op dit moment uw burgerlijke status?

❐ Gehuwd / samenwonend

❐ Gescheiden / uit elkaar gegaan

❐ Weduwe / weduwnaar / partner overleden

❐ Nooit gehuwd / nooit samengewoond

3. Wat is het hoogste opleidingsniveau dat u heeft afgerond?

❐ Lager onderwijs (of minder)

❐ Voortgezet onderwijs, of gelijkwaardig

❐ Middelbaar (beroeps-) onderwijs, of gelijkwaardig

❐ Universiteit, Hoger (beroeps-) onderwijs, of gelijkwaardig

4. Heeft u op dit moment betaald werk?

❐ Ja, voor ☐☐ uur per week in loondienst

❐ Ja, ik ben zelfstandig ondernemer voor ☐☐ uur per week

❐ Nee → ga verder met vraag 7

5. Wat is uw beroep of functie (op dit moment):

6. Welke veranderingen zijn er opgetreden in uw werksituatie als gevolg van de teruggekeerde kanker?

- ❒ Er is niets veranderd als gevolg van de teruggekeerde kanker
- ❒ Ik ben omgeschoold als gevolg van de teruggekeerde kanker
- ❒ Ik heb een andere functie gekregen als gevolg van de teruggekeerde kanker, vroeger was ik:
- ❒ Ik ben volledig gestopt met werken als gevolg van de teruggekeerde kanker
- ❒ Ik ben meer gaan werken als gevolg van de teruggekeerde kanker, namelijk ☐☐ uur meer
- ❒ Ik ben minder gaan werken als gevolg van de teruggekeerde kanker, namelijk ☐☐ uur minder
- ❒ Ik ben arbeidsongeschikt ten gevolge van de teruggekeerde kanker
- ❒ Ik ben vervroegd gepensioneerd als gevolg van de teruggekeerde kanker
- ❒ Ik ben ontslagen als gevolg van de teruggekeerde kanker

7. Indien u geen betaald werk heeft, welke van de volgende redenen is het meest op u van toepassing? Als u betaald werk heeft mag u deze vraag overslaan.

- ❒ Pensioen
- ❒ Scholier/student
- ❒ Werkloos
- ❒ Arbeidsongeschikt (WIA, WAO of WAJONG)
- ❒ Dagtaak aan zorg voor huishouden en eventueel kinderen
- ❒ Iets anders, namelijk

Vragen over uw gezondheid

Wij zijn geïnteresseerd in bepaalde dingen over u en uw gezondheid. Wilt u alle vragen zelf beantwoorden door één hokje aan te kruisen dat het meest op u van toepassing is. Er zijn geen "goede" of "foute" antwoorden.

	**Gedurende de afgelopen week:**	**Helemaal niet**	**Een beetje**	**Nogal**	**Heel erg**
8.	Heeft u moeite met het doen van inspannende activiteiten zoals het dragen van een zware boodschappentas of een koffer?	❒	❒	❒	❒
9.	Heeft u moeite met het maken van een lange wandeling?	❒	❒	❒	❒
10.	Heeft u moeite met het maken van een korte wandeling buitenshuis?	❒	❒	❒	❒
11.	Moet u overdag in bed of op een stoel blijven?	❒	❒	❒	❒
12.	Heeft u hulp nodig met eten, aankleden, uzelf wassen of naar het toilet gaan?	❒	❒	❒	❒
13.	Was u beperkt bij het doen van uw werk of andere dagelijkse bezigheden?	❒	❒	❒	❒
14.	Was u beperkt in het uitoefenen van uw hobby's of bij andere bezigheden die u in uw vrije tijd doet?	❒	❒	❒	❒
15.	Was u kortademig?	❒	❒	❒	❒
16.	Heeft u pijn gehad?	❒	❒	❒	❒
17.	Had u behoefte om te rusten?	❒	❒	❒	❒
18.	Heeft u moeite met slapen gehad?	❒	❒	❒	❒
19.	Heeft u zich slap gevoeld?	❒	❒	❒	❒
20.	Heeft u gebrek aan eetlust gehad?	❒	❒	❒	❒
21.	Heeft u zich misselijk gevoeld?	❒	❒	❒	❒
22.	Heeft u overgegeven?	❒	❒	❒	❒
23.	Had u last van obstipatie (was u verstopt)?	❒	❒	❒	❒
24.	Had u diarree?	❒	❒	❒	❒

	**Gedurende de <u>afgelopen week</u>:**	**Helemaal niet**	**Een beetje**	**Nogal**	**Heel erg**
25.	Was u moe?	❒	❒	❒	❒
26.	Heeft pijn u gehinderd in uw dagelijkse bezigheden?	❒	❒	❒	❒
27.	Heeft u moeite gehad met het concentreren op dingen zoals een krant lezen of televisiekijken?	❒	❒	❒	❒
28.	Voelde u zich gespannen?	❒	❒	❒	❒
29.	Maakte u zich zorgen?	❒	❒	❒	❒
30.	Voelde u zich prikkelbaar?	❒	❒	❒	❒
31.	Voelde u zich neerslachtig?	❒	❒	❒	❒
32.	Heeft u moeite gehad met het herinneren van dingen?	❒	❒	❒	❒
33.	Heeft uw lichamelijke toestand of medische behandeling uw familieleven in de weg gestaan?	❒	❒	❒	❒
34.	Heeft uw lichamelijke toestand of medische behandeling u belemmerd in uw sociale bezigheden?	❒	❒	❒	❒
35.	Heeft uw lichamelijke toestand of medische behandeling financiële moeilijkheden met zich meegebracht?	❒	❒	❒	❒

Wilt u voor de volgende vragen het getal tussen 1 en 7 aankruisen dat het meest op u van toepassing is

36. Hoe zou u uw <u>algehele gezondheid</u> gedurende de afgelopen week beoordelen?

Erg slecht					Uitstekend	
1	2	3	4	5	6	7
❒	❒	❒	❒	❒	❒	❒

37. Hoe zou u uw algehele <u>"kwaliteit van het leven"</u> gedurende de afgelopen week beoordelen?

Erg slecht					Uitstekend	
1	2	3	4	5	6	7
❒	❒	❒	❒	❒	❒	❒

Vragen over klachten

Soms melden patiënten dat ze de volgende symptomen of problemen ervaren. Wilt u alle vragen zelf beantwoorden door één hokje aan te kruisen dat het meest op u van toepassing is.

	**Gedurende de afgelopen week:**	**Helemaal niet**	**Een beetje**	**Nogal**	**Heel erg**
38.	Had u pijn in de maagstreek?	❒	❒	❒	❒
39.	Heeft u een vol gevoel in uw buik/maag gehad?	❒	❒	❒	❒
40.	Heeft u het gevoel gehad dat uw kleren te strak zaten?	❒	❒	❒	❒
41.	Is er iets veranderd aan uw stoelgang ten gevolge van uw ziekte of behandeling?	❒	❒	❒	❒
42.	Heeft u last van winderigheid?	❒	❒	❒	❒
43.	Heeft u het gevoel gehad te snel voldaan te zijn nadat u bent begonnen te eten?	❒	❒	❒	❒
44.	Heeft u indigestie of maagzuur gehad?	❒	❒	❒	❒
45.	Heeft u haaruitval gehad?	❒	❒	❒	❒
46.	Deze vraag alleen invullen indien u haaruitval heeft gehad: Had u last van haaruitval?	❒	❒	❒	❒
47.	Smaakten voedsel en drank anders dan gewoonlijk?	❒	❒	❒	❒
48.	Had u prikkelende handen of voeten?	❒	❒	❒	❒
49.	Heeft u een doof gevoel in uw vingers of tenen gehad?	❒	❒	❒	❒
50.	Heeft u zich zwak in uw armen of benen gevoeld?	❒	❒	❒	❒
51.	Heeft u pijn gehad in uw spieren of gewrichten?	❒	❒	❒	❒
52.	Heeft u problemen met uw gehoor gehad?	❒	❒	❒	❒
53.	Moest u vaak plassen?	❒	❒	❒	❒
54.	Heeft u huidproblemen gehad (bijv. jeukerig, droog)?	❒	❒	❒	❒
55.	Heeft u opvliegers (vapeurs) gehad?	❒	❒	❒	❒
56.	Heeft nachtzweten gehad?	❒	❒	❒	❒
57.	Voelde u zich lichamelijk minder aantrekkelijk ten gevolge van uw ziekte of behandeling?	❒	❒	❒	❒
58.	Voelde u zich ontevreden over uw lichaam als gevolg van de ziekte of behandeling?	❒	❒	❒	❒

	**Gedurende de afgelopen week:**	**Helemaal niet**	**Een beetje**	**Nogal**	**Heel erg**
59.	Hoe belastend is uw ziekte geweest voor u?	❐	❐	❐	❐
60.	Hoe belastend is uw behandeling geweest voor u?	❐	❐	❐	❐
61.	Heeft u zich zorgen gemaakt over uw gezondheid in de toekomst?	❐	❐	❐	❐

	**Gedurende de afgelopen 4 weken:**	**Helemaal niet**	**Een beetje**	**Nogal**	**Heel erg**	**Wil ik niet zeggen**
62.	Heeft u belangstelling voor seks gehad?	❐	❐	❐	❐	❐
63.	Bent u seksueel actief geweest?	❐	❐	❐	❐	❐
	De onderstaande vragen alleen beantwoorden indien u seksueel actief bent geweest gedurende de afgelopen 4 weken:					
64.	Zijn seksuele activiteiten plezierig voor u geweest?	❐	❐	❐	❐	❐
65.	Had u een droge vagina tijdens de gemeenschap?	❐	❐	❐	❐	❐

Vragen over de kwaliteit van leven
Wij willen graag weten in welke mate de behandeling invloed heeft op uw kwaliteit van leven. De volgende vragen gaan over uw huidige gezondheid. Kruist u alstublieft één hokje per vraag aan met de keuze welke het best past bij uw gezondheid VANDAAG.

66. Mobiliteit
- ❐ Ik heb geen problemen met lopen
- ❐ Ik heb een beetje problemen met lopen
- ❐ Ik heb matige problemen met lopen
- ❐ Ik heb ernstige problemen met lopen
- ❐ Ik ben niet in staat om te lopen

67. Zelfzorg
- ❐ Ik heb geen problemen met mijzelf wassen of aankleden
- ❐ Ik heb een beetje problemen met mijzelf wassen of aankleden
- ❐ Ik heb matige problemen met mijzelf wassen of aankleden
- ❐ Ik heb ernstige problemen met mijzelf wassen of aankleden
- ❐ Ik ben niet in staat om mijzelf te wassen of aan te kleden

68. Dagelijkse activiteiten *(bijvoorbeeld werk, studie, huishouden, gezins- en vrijetijdsactiviteiten)*
- ❐ Ik heb geen problemen met mijn dagelijks activiteiten
- ❐ Ik heb een beetje problemen met mijn dagelijkse activiteiten
- ❐ Ik heb matige problemen met mijn dagelijkse activiteiten
- ❐ Ik heb ernstige problemen met mijn dagelijkse activiteiten
- ❐ Ik ben niet in staat mijn dagelijkse activiteiten uit te voeren

69. Pijn/ongemak
- ❒ Ik heb geen pijn of ongemak
- ❒ Ik heb een beetje pijn of ongemak
- ❒ Ik heb matige pijn of ongemak
- ❒ Ik heb ernstige pijn of ongemak
- ❒ Ik heb extreme pijn of ongemak

70. Angst/somberheid
- ❒ Ik ben niet angstig of somber
- ❒ Ik ben een beetje angstig of somber
- ❒ Ik ben matig angstig of somber
- ❒ Ik ben erg angstig of somber
- ❒ Ik ben extreem angstig of somber

71. Gezondheidstoestand

We zouden graag willen weten hoe goed of hoe slecht volgens u uw eigen gezondheidstoestand VANDAAG is. Om u te helpen bij het aangeven hoe goed of hoe slecht uw gezondheidstoestand is, hebben we een meetschaal (te vergelijken met een thermometer) gemaakt. Deze meetschaal loopt van 0 tot 100.

- 100 staat voor de beste gezondheid die u zich kunt voorstellen.
- 0 staat voor de slechtste gezondheid die u zich kunt voorstellen.

We willen u vragen om op deze meetschaal met een X aan te geven hoe goed of hoe slecht uw eigen gezondheidstoestand VANDAAG is.

Noteer vervolgens het getal waarbij u de X heeft geplaatst in onderstaand vakje.

Uw gezondheid vandaag:

De beste gezondheid die u zich kunt voorstellen

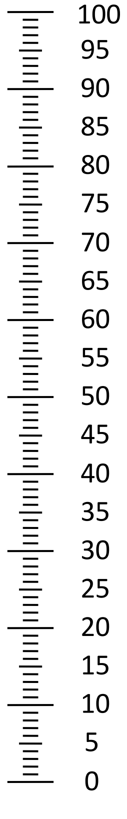

De slechtste gezondheid die u zich kunt voorstellen

Aanvullende vragen over klachten

Wanneer mensen behandeld worden voor kanker ervaren ze soms verschillende symptomen en bijwerkingen. Selecteer voor elke vraag het antwoord dat het beste uw ervaring tussen de afgelopen 7 dagen beschrijft. Hoewel sommige vragen op elkaar kunnen lijken, is het toch belangrijk dat u alle vragen invult.

**72. Droge mond**<br><br>Hoe ERG was uw DROGE MOND op het SLECHTSTE MOMENT in de afgelopen 7 dagen?				
❒ Helemaal niet	❒ Een beetje	❒ Matig	❒ Erg	❒ Heel erg

**73. Moeite met slikken**<br><br>Hoe ERG was de MOEITE die u had MET SLIKKEN op het SLECHTSTE MOMENT in de afgelopen 7 dagen?				
❒ Helemaal niet	❒ Een beetje	❒ Matig	❒ Erg	❒ Heel erg

**74. Zweertjes in de mond/keel (aften)**<br><br>Hoe ERG waren de ZWEERTJES IN UW MOND OF KEEL (AFTEN) op het SLECHTSTE MOMENT in de afgelopen 7 dagen?				
❒ Helemaal niet	❒ Een beetje	❒ Matig	❒ Erg	❒ Heel erg

**75. Zweertjes in de mond/keel (aften)**<br><br>In welke mate hebben de ZWEERTJES IN UW MOND OF KEEL (AFTEN) uw gebruikelijke of dagelijkse activiteiten BELEMMERD in de afgelopen 7 dagen?				
❒ Helemaal niet	❒ Een beetje	❒ Enigszins	❒ Redelijk wat	❒ Zeer veel

**76. Kloofjes in de mondhoeken (cheilose/chelitis)**<br><br>Hoe ERG waren de KLOOFJES IN UW MONDHOEKEN op het SLECHTSTE MOMENT in de afgelopen 7 dagen?				
❒ Helemaal niet	❒ Een beetje	❒ Matig	❒ Erg	❒ Heel erg

**77. Veranderingen in stemkwaliteit**<br><br>Hebt u VERANDERINGEN IN UW STEM gehad in de afgelopen 7 dagen?	
❒ Ja	❒ Nee

**78. Heesheid**<br><br>Hoe ERG was uw HESE STEM op het SLECHTSTE MOMENT in de afgelopen 7 dagen?				
❒ Helemaal niet	❒ Een beetje	❒ Matig	❒ Erg	❒ Heel erg

**79. Veranderingen in smaak**<br><br>Hoe ERG waren uw PROBLEMEN MET DE SMAAK VAN ETEN OF DRINKEN op het SLECHTSTE MOMENT in de afgelopen 7 dagen?				
❒ Helemaal niet	❒ Een beetje	❒ Matig	❒ Erg	❒ Heel erg

**80. Verminderde eetlust**<br><br>Hoe ERG was uw VERMINDERDE EETLUST op het SLECHTSTE MOMENT in de afgelopen 7 dagen?				
❒ Helemaal niet	❒ Een beetje	❒ Matig	❒ Erg	❒ Heel erg

**81. Verminderde eetlust**<br><br>In welke mate heeft VERMINDERDE EETLUST uw gebruikelijke of dagelijkse activiteiten BELEMMERD in de afgelopen 7 dagen?				
❒ Helemaal niet	❒ Een beetje	❒ Enigszins	❒ Redelijk wat	❒ Zeer veel

**82. Misselijkheid**<br><br>Hoe VAAK bent u MISSELIJK geweest in de afgelopen 7 dagen?				
❒ Nooit	❒ Zelden	❒ Soms	❒ Vaak	❒ Bijna voortdurend

**83. Misselijkheid**<br><br>Hoe ERG was uw MISSELIJKHEID op het SLECHTSTE MOMENT in de afgelopen 7 dagen?				
❒ Helemaal niet	❒ Een beetje	❒ Matig	❒ Erg	❒ Heel erg

**84. Braken**<br><br>Hoe VAAK hebt u GEBRAAKT in de afgelopen 7 dagen?				
❒ Nooit	❒ Zelden	❒ Soms	❒ Vaak	❒ Bijna voortdurend

**85. Braken**<br><br>Hoe ERG was uw BRAKEN op het SLECHTSTE MOMENT in de afgelopen 7 dagen?				
❒ Helemaal niet	❒ Een beetje	❒ Matig	❒ Erg	❒ Heel erg

**86. Brandend maagzuur**<br><br>Hoe VAAK hebt u BRANDEND MAAGZUUR gehad in de afgelopen 7 dagen?				
❒ Nooit	❒ Zelden	❒ Soms	❒ Vaak	❒ Bijna voortdurend

**87. Brandend maagzuur**<br><br>Hoe ERG was uw BRANDEND MAAGZUUR op het SLECHTSTE MOMENT in de afgelopen 7 dagen?				
❒ Helemaal niet	❒ Een beetje	❒ Matig	❒ Erg	❒ Heel erg

**88. Winderigheid**<br><br>Hebt u MEER WINDEN MOETEN LATEN (FLATULENTIE) in de afgelopen 7 dagen?	
❒ Ja	❒ Nee

**89. Opgeblazen gevoel**<br><br>Hoe VAAK hebt u een OPGEBLAZEN GEVOEL IN UW BUIK gehad in de afgelopen 7 dagen?				
❒ Nooit	❒ Zelden	❒ Soms	❒ Vaak	❒ Bijna voortdurend

**90. Opgeblazen gevoel**<br><br>Hoe ERG was uw OPGEBLAZEN GEVOEL IN UW BUIK op het SLECHTSTE MOMENT in de afgelopen 7 dagen?				
❒ Helemaal niet	❒ Een beetje	❒ Matig	❒ Erg	❒ Heel erg

**91. De hik**<br><br>Hoe VAAK hebt u DE HIK GEHAD in de afgelopen 7 dagen?				
❒ Nooit	❒ Zelden	❒ Soms	❒ Vaak	❒ Bijna voortdurend

**92. De hik**<br><br>Hoe ERG was uw HIK op het SLECHTSTE MOMENT in de afgelopen 7 dagen?				
❒ Helemaal niet	❒ Een beetje	❒ Matig	❒ Erg	❒ Heel erg

**93. Verstopping**<br><br>Hoe ERG was uw VERSTOPPING op het SLECHTSTE MOMENT in de afgelopen 7 dagen?				
❒ Helemaal niet	❒ Een beetje	❒ Matig	❒ Erg	❒ Heel erg

94. Diarree

Hoe VAAK hebt u DUNNE OF WATERIGE STOELGANG (DIARREE) gehad in de afgelopen 7 dagen?

❐ Nooit	❐ Zelden	❐ Soms	❐ Vaak	❐ Bijna voortdurend

95. Buikpijn

Hoe VAAK hebt u BUIKPIJN gehad in de afgelopen 7 dagen?

❐ Nooit	❐ Zelden	❐ Soms	❐ Vaak	❐ Bijna voortdurend

96. Buikpijn

Hoe ERG was uw BUIKPIJN op het SLECHTSTE MOMENT in de afgelopen 7 dagen?

❐ Helemaal niet	❐ Een beetje	❐ Matig	❐ Erg	❐ Heel erg

97. Buikpijn

In welke mate heeft de BUIKPIJN uw gebruikelijke of dagelijkse activiteiten BELEMMERD in de afgelopen 7 dagen?

❐ Helemaal niet	❐ Een beetje	❐ Enigszins	❐ Redelijk wat	❐ Zeer veel

98. Ontlastingsincontinentie

Hoe VAAK hebt u CONTROLEVERLIES OVER UW ONTLASTING gehad in de afgelopen 7 dagen?

❐ Nooit	❐ Zelden	❐ Soms	❐ Vaak	❐ Bijna voortdurend

99. Ontlastingsincontinentie

In welke mate heeft het CONTROLEVERLIES OVER UW ONTLASTING uw gebruikelijke of dagelijkse activiteiten BELEMMERD in de afgelopen 7 dagen?

❐ Helemaal niet	❐ Een beetje	❐ Enigszins	❐ Redelijk wat	❐ Zeer veel

100. Kortademigheid

Hoe ERG was uw KORTADEMIGHEID op het SLECHTSTE MOMENT in de afgelopen 7 dagen?

❐ Helemaal niet	❐ Een beetje	❐ Matig	❐ Erg	❐ Heel erg

101. Kortademigheid

In welke mate heeft uw KORTADEMIGHEID uw gebruikelijke of dagelijkse activiteiten BELEMMERD in de afgelopen 7 dagen?

❐ Helemaal niet	❐ Een beetje	❐ Enigszins	❐ Redelijk wat	❐ Zeer veel

102. Hoest

Hoe ERG was uw HOEST op het SLECHTSTE MOMENT in de afgelopen 7 dagen?

❒ Helemaal niet ❒ Een beetje ❒ Matig ❒ Erg ❒ Heel erg

103. Hoest

In welke mate heeft HOESTEN uw gebruikelijke of dagelijkse activiteiten BELEMMERD in de afgelopen 7 dagen?

❒ Helemaal niet ❒ Een beetje ❒ Enigszins ❒ Redelijk wat ❒ Zeer veel

104. Piepende ademhaling

Hoe ERG was uw PIEPENDE ADEMHALING (FLUITEND GELUID IN DE BORST BIJ HET ADEMHALEN) op het SLECHTSTE MOMENT in de afgelopen 7 dagen?

❒ Helemaal niet ❒ Een beetje ❒ Matig ❒ Erg ❒ Heel erg

105. Zwelling

Hoe VAAK hebt u een GEZWOLLEN ARM OF BEEN gehad in de afgelopen 7 dagen?

❒ Nooit ❒ Zelden ❒ Soms ❒ Vaak ❒ Bijna voortdurend

106. Zwelling

Hoe ERG was de ZWELLING IN ARM OF BEEN op het SLECHTSTE MOMENT in de afgelopen 7 dagen?

❒ Helemaal niet ❒ Een beetje ❒ Matig ❒ Erg ❒ Heel erg

107. Zwelling

In welke mate heeft de ZWELLING IN ARM OF BEEN uw gebruikelijke of dagelijkse activiteiten BELEMMERD in de afgelopen 7 dagen?

❒ Helemaal niet ❒ Een beetje ❒ Enigszins ❒ Redelijk wat ❒ Zeer veel

108. Hartkloppingen

Hoe VAAK hebt u een BONZENDE OF JAGENDE HARTSLAG (HARTKLOPPINGEN) gehad in de afgelopen 7 dagen?

❒ Nooit ❒ Zelden ❒ Soms ❒ Vaak ❒ Bijna voortdurend

109. Hartkloppingen

Hoe ERG was uw BONZENDE OF JAGENDE HARTSLAG (HARTKLOPPINGEN) op het SLECHTSTE MOMENT in de afgelopen 7 dagen?

❒ Helemaal niet ❒ Een beetje ❒ Matig ❒ Erg ❒ Heel erg

**110. Huiduitslag**<br><br>Hebt u enige vorm van HUIDUITSLAG gehad in de afgelopen 7 dagen?	
❒ Ja	❒ Nee

**111. Droge huid**<br><br>Hoe ERG was uw DROGE HUID op het SLECHTSTE MOMENT in de afgelopen 7 dagen?				
❒ Helemaal niet	❒ Een beetje	❒ Matig	❒ Erg	❒ Heel erg

**112. Acne**<br><br>Hoe ERG was/waren uw ACNE OF PUISTJES OP HET GEZICHT OF DE BORST op het SLECHTSTE MOMENT in de afgelopen 7 dagen?				
❒ Helemaal niet	❒ Een beetje	❒ Matig	❒ Erg	❒ Heel erg

**113. Haaruitval**<br><br>Hebt u HAARUITVAL gehad in de afgelopen 7 dagen?				
❒ Helemaal niet	❒ Een beetje	❒ Enigszins	❒ Redelijk wat	❒ Zeer veel

**114. Jeuk**<br><br>Hoe ERG was uw JEUKENDE HUID op het SLECHTSTE MOMENT in de afgelopen 7 dagen?				
❒ Helemaal niet	❒ Een beetje	❒ Matig	❒ Erg	❒ Heel erg

**115. Netelroos**<br><br>Hebt u NETELROOS (JEUKERIGE RODE BULTJES OP DE HUID) gehad in de afgelopen 7 dagen?	
❒ Ja	❒ Nee

**116. Hand-voetsyndroom**<br><br>Hoe ERG was uw HAND-VOETSYNDROOM (EEN UITSLAG OP DE HANDEN OF VOETEN DIE KLOOFJES, AFSCHILFEREN, ROODHEID OF PIJN KAN VEROORZAKEN) op het SLECHTSTE MOMENT in de afgelopen 7 dagen?				
❒ Helemaal niet	❒ Een beetje	❒ Matig	❒ Erg	❒ Heel erg

**117. Nagelverlies**<br><br>Hebt u VINGERNAGELS OF TEENNAGELS VERLOREN in de afgelopen 7 dagen?	
❒ Ja	❒ Nee

**118. Geribbelde nagels**<br><br>Hebt u RIBBELS OF BULTJES OP UW VINGERNAGELS OF TEENNAGELS gehad in de afgelopen 7 dagen?	
❒ Ja	❒ Nee

**119. Nagelverkleuring**<br><br>Hebt u een VERANDERING IN DE KLEUR VAN UW VINGERNAGELS OF TEENNAGELS gehad in de afgelopen 7 dagen?	
❒ Ja	❒ Nee

**120. Gevoeligheid voor zonlicht**<br><br>Is de GEVOELIGHEID VAN UW HUID VOOR ZONLICHT TOEGENOMEN in de afgelopen 7 dagen?	
❒ Ja	❒ Nee

**121. Doorlig-/drukwonden**<br><br>Hebt u DOORLIGWONDEN gehad in de afgelopen 7 dagen?	
❒ Ja	❒ Nee

**122. Huidreactie op bestraling**<br><br>Hoe ERG waren de BRANDWONDEN OP UW HUID DOOR BESTRALING op het SLECHTSTE MOMENT in de afgelopen 7 dagen?				
❒ Niet van toepassing				
❒ Helemaal niet	❒ Een beetje	❒ Matig	❒ Erg	❒ Heel erg

**123. Huidverdonkering**<br><br>Hebt u ONGEWONE DONKERE VERKLEURING VAN DE HUID gehad in de afgelopen 7 dagen?	
❒ Ja	❒ Nee

**124. Striemen**<br><br>Hebt u STRIEMEN (STRIAE) gehad in de afgelopen 7 dagen?	
❒ Ja	❒ Nee

**125. Gevoelloosheid en tintelen**<br><br>Hoe ERG was uw GEVOELLOOSHEID OF HET TINTELEN IN UW HANDEN OF VOETEN op het SLECHTSTE MOMENT in de afgelopen 7 dagen?				
❒ Helemaal niet	❒ Een beetje	❒ Matig	❒ Erg	❒ Heel erg

**126. Gevoelloosheid en tintelen**<br><br>In welke mate heeft de GEVOELLOOSHEID OF HET TINTELEN IN UW HANDEN OF VOETEN uw gebruikelijke of dagelijkse activiteiten BELEMMERD in de afgelopen 7 dagen?				
❒ Helemaal niet	❒ Een beetje	❒ Enigszins	❒ Redelijk wat	❒ Zeer veel

**127. Duizeligheid**<br><br>Hoe ERG was uw DUIZELIGHEID op het SLECHTSTE MOMENT in de afgelopen 7 dagen?				
❒ Helemaal niet	❒ Een beetje	❒ Matig	❒ Erg	❒ Heel erg

**128. Duizeligheid**<br><br>In welke mate heeft DUIZELIGHEID uw gebruikelijke of dagelijkse activiteiten BELEMMERD in de afgelopen 7 dagen?				
❒ Helemaal niet	❒ Een beetje	❒ Enigszins	❒ Redelijk wat	❒ Zeer veel

**129. Wazig zicht**<br><br>Hoe ERG was uw WAZIG ZICHT op het SLECHTSTE MOMENT in de afgelopen 7 dagen?				
❒ Helemaal niet	❒ Een beetje	❒ Matig	❒ Erg	❒ Heel erg

**130. Wazig zicht**<br><br>In welke mate heeft WAZIG ZICHT uw gebruikelijke of dagelijkse activiteiten BELEMMERD in de afgelopen 7 dagen?				
❒ Helemaal niet	❒ Een beetje	❒ Enigszins	❒ Redelijk wat	❒ Zeer veel

**131. Lichtflitsen**<br><br>Hebt u LICHTFLITSEN VÓÓR UW OGEN gehad in de afgelopen 7 dagen?	
❒ Ja	❒ Nee

**132. Visuele floaters**<br><br>Hebt u ZWEVENDE VLEKJES OF SLIERTJES DIE VÓÓR UW OGEN BEWEGEN gehad in de afgelopen 7 dagen?	
❐ Ja	❐ Nee

**133. Tranende ogen**<br><br>Hoe ERG waren uw TRANENDE OGEN op het SLECHTSTE MOMENT in de afgelopen 7 dagen?				
❐ Helemaal niet	❐ Een beetje	❐ Matig	❐ Erg	❐ Heel erg

**134. Tranende ogen**<br><br>In welke mate hebben TRANENDE OGEN uw gebruikelijke of dagelijkse activiteiten BELEMMERD in de afgelopen 7 dagen?				
❐ Helemaal niet	❐ Een beetje	❐ Enigszins	❐ Redelijk wat	❐ Zeer veel

**135. Oorsuizen**<br><br>Hoe ERG was UW OORSUIZEN op het SLECHTSTE MOMENT in de afgelopen 7 dagen?				
❐ Helemaal niet	❐ Een beetje	❐ Matig	❐ Erg	❐ Heel erg

**136. Concentratie**<br><br>Hoe ERG waren uw CONCENTRATIEPROBLEMEN op het SLECHTSTE MOMENT in de afgelopen 7 dagen?				
❐ Helemaal niet	❐ Een beetje	❐ Matig	❐ Erg	❐ Heel erg

**137. Concentratie**<br><br>In welke mate hebben CONCENTRATIEPROBLEMEN uw gebruikelijke of dagelijkse activiteiten BELEMMERD in de afgelopen 7 dagen?				
❐ Helemaal niet	❐ Een beetje	❐ Enigszins	❐ Redelijk wat	❐ Zeer veel

**138. Geheugen**<br><br>Hoe ERG waren uw GEHEUGENPROBLEMEN op het SLECHTSTE MOMENT in de afgelopen 7 dagen?				
❐ Helemaal niet	❐ Een beetje	❐ Matig	❐ Erg	❐ Heel erg

**139. Geheugen**				
In welke mate hebben GEHEUGENPROBLEMEN uw gebruikelijke of dagelijkse activiteiten BELEMMERD in de afgelopen 7 dagen?				
❒ Helemaal niet	❒ Een beetje	❒ Enigszins	❒ Redelijk wat	❒ Zeer veel

**140. Algemene pijn**				
Hoe VAAK hebt u PIJN gehad in de afgelopen 7 dagen?				
❒ Nooit	❒ Zelden	❒ Soms	❒ Vaak	❒ Bijna voortdurend

**141. Algemene pijn**				
Hoe ERG was uw PIJN op het SLECHTSTE MOMENT in de afgelopen 7 dagen?				
❒ Helemaal niet	❒ Een beetje	❒ Matig	❒ Erg	❒ Heel erg

**142. Algemene pijn**				
In welke mate heeft PIJN uw gebruikelijke of dagelijkse activiteiten BELEMMERD in de afgelopen 7 dagen?				
❒ Helemaal niet	❒ Een beetje	❒ Enigszins	❒ Redelijk wat	❒ Zeer veel

**143. Hoofdpijn**				
Hoe VAAK hebt u HOOFDPIJN gehad in de afgelopen 7 dagen?				
❒ Nooit	❒ Zelden	❒ Soms	❒ Vaak	❒ Bijna voortdurend

**144. Hoofdpijn**				
Hoe ERG was uw HOOFDPIJN op het SLECHTSTE MOMENT in de afgelopen 7 dagen?				
❒ Helemaal niet	❒ Een beetje	❒ Matig	❒ Erg	❒ Heel erg

**145. Hoofdpijn**				
In welke mate heeft uw HOOFDPIJN uw gebruikelijke of dagelijkse activiteiten BELEMMERD in de afgelopen 7 dagen?				
❒ Helemaal niet	❒ Een beetje	❒ Enigszins	❒ Redelijk wat	❒ Zeer veel

**146. Spierpijn**				
Hoe VAAK hebt u SPIERPIJN gehad in de afgelopen 7 dagen?				
❒ Nooit	❒ Zelden	❒ Soms	❒ Vaak	❒ Bijna voortdurend

**147. Spierpijn**<br><br>Hoe ERG was uw SPIERPIJN op het SLECHTSTE MOMENT in de afgelopen 7 dagen?				
❒ Helemaal niet	❒ Een beetje	❒ Matig	❒ Erg	❒ Heel erg

**148. Spierpijn**<br><br>In welke mate heeft uw SPIERPIJN uw gebruikelijke of dagelijkse activiteiten BELEMMERD in de afgelopen 7 dagen?				
❒ Helemaal niet	❒ Een beetje	❒ Enigszins	❒ Redelijk wat	❒ Zeer veel

**149. Gewrichtspijn**<br><br>Hoe VAAK hebt u PIJNLIJKE GEWRICHTEN (ZOALS ELLEBOGEN, KNIEËN, SCHOUDERS) gehad in de afgelopen 7 dagen?				
❒ Nooit	❒ Zelden	❒ Soms	❒ Vaak	❒ Bijna voortdurend

**150. Gewrichtspijn**<br><br>Hoe ERG waren uw PIJNLIJKE GEWRICHTEN (ZOALS ELLEBOGEN, KNIEËN, SCHOUDERS) op het SLECHTSTE MOMENT in de afgelopen 7 dagen?				
❒ Helemaal niet	❒ Een beetje	❒ Matig	❒ Erg	❒ Heel erg

**151. Gewrichtspijn**<br><br>In welke mate hebben uw PIJNLIJKE GEWRICHTEN (ZOALS ELLEBOGEN, KNIEËN, SCHOUDERS) uw gebruikelijke of dagelijkse activiteiten BELEMMERD in de afgelopen 7 dagen?				
❒ Helemaal niet	❒ Een beetje	❒ Enigszins	❒ Redelijk wat	❒ Zeer veel

**152. Slapeloosheid**<br><br>Hoe ERG was uw SLAPELOOSHEID (WAARONDER MOEITE MET IN SLAAP VALLEN, DOORSLAPEN, OF VROEG WAKKER WORDEN) op het SLECHTSTE MOMENT in de afgelopen 7 dagen?				
❒ Helemaal niet	❒ Een beetje	❒ Matig	❒ Erg	❒ Heel erg

**153. Slapeloosheid**<br><br>In welke mate heeft uw SLAPELOOSHEID (WAARONDER MOEITE MET IN SLAAP VALLEN, DOORSLAPEN, OF VROEG WAKKER WORDEN) uw gebruikelijke of dagelijkse activiteiten BELEMMERD in de afgelopen 7 dagen?				
❒ Helemaal niet	❒ Een beetje	❒ Enigszins	❒ Redelijk wat	❒ Zeer veel

**153. Vermoeidheid**<br><br>Hoe ERG was uw VERMOEIDHEID OF GEBREK AAN ENERGIE op het SLECHTSTE MOMENT in de afgelopen 7 dagen?				
❒ Helemaal niet	❒ Een beetje	❒ Matig	❒ Erg	❒ Heel erg

**154. Vermoeidheid**<br><br>In welke mate heeft uw VERMOEIDHEID OF GEBREK AAN ENERGIE uw gebruikelijke of dagelijkse activiteiten BELEMMERD in de afgelopen 7 dagen?				
❒ Helemaal niet	❒ Een beetje	❒ Enigszins	❒ Redelijk wat	❒ Zeer veel

**155. Angst**<br><br>Hoe VAAK bent u ANGSTIG geweest in de afgelopen 7 dagen?				
❒ Nooit	❒ Zelden	❒ Soms	❒ Vaak	❒ Bijna voortdurend

**156. Angst**<br><br>Hoe ERG was uw ANGST op het SLECHTSTE MOMENT in de afgelopen 7 dagen?				
❒ Helemaal niet	❒ Een beetje	❒ Matig	❒ Erg	❒ Heel erg

**157. Angst**<br><br>In welke mate heeft ANGST uw gebruikelijke of dagelijkse activiteiten BELEMMERD in de afgelopen 7 dagen?				
❒ Helemaal niet	❒ Een beetje	❒ Enigszins	❒ Redelijk wat	❒ Zeer veel

**158. Ontmoedigd**<br><br>Hoe VAAK hebt u GEVOELD DAT NIETS U KON OPVROLIJKEN in de afgelopen 7 dagen?				
❒ Nooit	❒ Zelden	❒ Soms	❒ Vaak	❒ Bijna voortdurend

**159. Ontmoedigd**<br><br>Hoe ERG was het GEVOEL DAT NIETS U KON OPVROLIJKEN op het SLECHTSTE MOMENT in de afgelopen 7 dagen?				
❒ Helemaal niet	❒ Een beetje	❒ Matig	❒ Erg	❒ Heel erg

**160. Ontmoedigd**<br><br>In welke mate heeft het GEVOEL DAT NIETS U KON OPVROLIJKEN uw gebruikelijke of dagelijkse activiteiten BELEMMERD in de afgelopen 7 dagen?				
❒ Helemaal niet	❒ Een beetje	❒ Enigszins	❒ Redelijk wat	❒ Zeer veel

**161. Verdrietig**				
Hoe VAAK hebt u zich VERDRIETIG OF ONGELUKKIG GEVOELD in de afgelopen 7 dagen?				
❒ Nooit	❒ Zelden	❒ Soms	❒ Vaak	❒ Bijna voortdurend

**162. Verdrietig**				
Hoe ERG waren uw VERDRIETIGE OF ONGELUKKIGE GEVOELENS op het SLECHTSTE MOMENT in de afgelopen 7 dagen?				
❒ Helemaal niet	❒ Een beetje	❒ Matig	❒ Erg	❒ Heel erg

**163. Verdrietig**				
In welke mate hebben VERDRIETIGE OF ONGELUKKIGE GEVOELENS uw gebruikelijke of dagelijkse activiteiten BELEMMERD in de afgelopen 7 dagen?				
❒ Helemaal niet	❒ Een beetje	❒ Enigszins	❒ Redelijk wat	❒ Zeer veel

**164. Onregelmatige menstruatie/vaginale bloeding**		
Hebt u ONREGELMATIGE MENSTRUATIE gehad in de afgelopen 7 dagen?		
❒ Ja	❒ Nee	❒ Niet van toepassing

**165. Overgeslagen verwachte menstruatie**		
Hebt u een VERWACHTE MENSTRUATIE OVERGESLAGEN in de afgelopen 7 dagen?		
❒ Ja	❒ Nee	❒ Niet van toepassing

**166. Vaginale afscheiding**				
Hebt u ONGEWONE VAGINALE AFSCHEIDING gehad in de afgelopen 7 dagen?				
❒ Helemaal niet	❒ Een beetje	❒ Enigszins	❒ Redelijk wat	❒ Zeer veel

**167. Vaginale droogheid**				
Hoe ERG was uw VAGINALE DROOGHEID op het SLECHTSTE MOMENT in de afgelopen 7 dagen?				
❒ Helemaal niet	❒ Een beetje	❒ Matig	❒ Erg	❒ Heel erg

**168. Pijn bij het plassen**				
Hoe ERG was uw PIJN OF BRANDENDE GEVOEL BIJ HET PLASSEN op het SLECHTSTE MOMENT in de afgelopen 7 dagen?				
❒ Helemaal niet	❒ Een beetje	❒ Matig	❒ Erg	❒ Heel erg

**169. Urinaire aandrang**<br><br>Hoe VAAK hebt u een DRANG OM PLOTSELING TE MOETEN PLASSEN gevoeld in de afgelopen 7 dagen?				
❒ Nooit	❒ Zelden	❒ Soms	❒ Vaak	❒ Bijna voortdurend

**170. Urinaire aandrang**<br><br>In welke mate heeft de DRANG OM PLOTSELING TE MOETEN PLASSEN uw gebruikelijke of dagelijkse activiteiten BELEMMERD in de afgelopen 7 dagen?				
❒ Helemaal niet	❒ Een beetje	❒ Enigszins	❒ Redelijk wat	❒ Zeer veel

**171. Urinaire frequentie**<br><br>Waren er momenten waarop u VAAK MOEST PLASSEN in de afgelopen 7 dagen?				
❒ Nooit	❒ Zelden	❒ Soms	❒ Vaak	❒ Bijna voortdurend

**172. Urinaire frequentie**<br><br>In welke mate heeft VAAK MOETEN PLASSEN uw gebruikelijke of dagelijkse activiteiten BELEMMERD in de afgelopen 7 dagen?				
❒ Helemaal niet	❒ Een beetje	❒ Enigszins	❒ Redelijk wat	❒ Zeer veel

**173. Verandering in gebruikelijke kleur van urine**<br><br>Is de KLEUR VAN UW URINE VERANDERD in de afgelopen 7 dagen?	
❒ Ja	❒ Nee

**174. Urinaire incontinentie**<br><br>Hoe VAAK hebt u VERLIES VAN CONTROLE OVER UW URINE (URINEVERLIES) gehad in de afgelopen 7 dagen?				
❒ Nooit	❒ Zelden	❒ Soms	❒ Vaak	❒ Bijna voortdurend

**175. Urinaire incontinentie**<br><br>In welke mate heeft het VERLIES VAN CONTROLE OVER UW URINE (URINEVERLIES) uw gebruikelijke of dagelijkse activiteiten BELEMMERD in de afgelopen 7 dagen?				
❒ Helemaal niet	❒ Een beetje	❒ Enigszins	❒ Redelijk wat	❒ Zeer veel

176. Verminderd libido

Hoe ERG was uw VERMINDERD SEKSUEEL VERLANGEN op het SLECHTSTE MOMENT in de afgelopen 7 dagen?

❒ Niet seksueel actief ❒ Antwoord liever niet
❒ Helemaal niet ❒ Een beetje ❒ Matig ❒ Erg ❒ Heel erg

177. Uitgesteld orgasme

Hebt u het gevoel gehad dat het TE LANG DUURDE OM EEN ORGASME TE KRIJGEN OF KLAAR TE KOMEN in de afgelopen 7 dagen?

❒ Niet seksueel actief ❒ Antwoord liever niet
❒ Ja ❒ Nee

178. Niet in staat een orgasme te hebben

Was het ONMOGELIJK EEN ORGASME TE KRIJGEN OF KLAAR TE KOMEN in de afgelopen 7 dagen?

❒ Niet seksueel actief ❒ Antwoord liever niet
❒ Ja ❒ Nee

179. Pijn bij geslachtsgemeenschap

Hoe ERG was de PIJN TIJDENS VAGINALE SEKS op het SLECHTSTE MOMENT in de afgelopen 7 dagen?

❒ Niet seksueel actief ❒ Antwoord liever niet
❒ Helemaal niet ❒ Een beetje ❒ Matig ❒ Erg ❒ Heel erg

180. Gezwollen en gevoelige borsten

Hoe ERG was de ZWELLING OF GEVOELIGHEID IN UW BORSTEN op het SLECHTSTE MOMENT in de afgelopen 7 dagen?

❒ Helemaal niet ❒ Een beetje ❒ Matig ❒ Erg ❒ Heel erg

181. Blauwe plekken

Kreeg u GEMAKKELIJK BLAUWE PLEKKEN in de afgelopen 7 dagen?

❒ Ja ❒ Nee

182. Koude rillingen

Hoe VAAK hebt u GEBIBBERD OF KOUDE RILLINGEN gehad in de afgelopen 7 dagen?

❒ Nooit ❒ Zelden ❒ Soms ❒ Vaak ❒ Bijna voortdurend

**183. Koude rillingen**<br><br>Hoe ERG was het BIBBEREN OF DE KOUDE RILLINGEN op het SLECHTSTE MOMENT in de afgelopen 7 dagen?				
❒ Helemaal niet	❒ Een beetje	❒ Matig	❒ Erg	❒ Heel erg

**184. Toegenomen transpiratie**<br><br>Hoe VAAK hebt u ONVERWACHT OF OVERMATIG GEZWEET OVERDAG OF 'S NACHTS (NIET IN VERBAND MET OPVLIEGERS) in de afgelopen 7 dagen?				
❒ Nooit	❒ Zelden	❒ Soms	❒ Vaak	❒ Bijna voortdurend

**185. Toegenomen transpiratie**<br><br>Hoe ERG was het ONVERWACHT OF OVERMATIG ZWETEN OVERDAG OF 'S NACHTS (NIET IN VERBAND MET OPVLIEGERS) op het SLECHTSTE MOMENT in de afgelopen 7 dagen?				
❒ Helemaal niet	❒ Een beetje	❒ Matig	❒ Erg	❒ Heel erg

**186. Afgenomen transpiratie**<br><br>Hebt u een ONVERWACHTE AFNAME VAN ZWETEN gehad in de afgelopen 7 dagen?	
❒ Ja	❒ Nee

**187. Opvliegers**<br><br>Hoe VAAK hebt u OPVLIEGERS gehad in de afgelopen 7 dagen?				
❒ Nooit	❒ Zelden	❒ Soms	❒ Vaak	❒ Bijna voortdurend

**188. Opvliegers**<br><br>Hoe ERG waren uw OPVLIEGERS op het SLECHTSTE MOMENT in de afgelopen 7 dagen?				
❒ Helemaal niet	❒ Een beetje	❒ Matig	❒ Erg	❒ Heel erg

**189. Bloedneus**<br><br>Hoe VAAK hebt u EEN BLOEDNEUS gehad in de afgelopen 7 dagen?				
❒ Nooit	❒ Zelden	❒ Soms	❒ Vaak	❒ Bijna voortdurend

**190. Bloedneus**<br><br>Hoe ERG waren uw NEUSBLOEDINGEN op het SLECHTSTE MOMENT in de afgelopen 7 dagen?				
❒ Helemaal niet	❒ Een beetje	❒ Matig	❒ Erg	❒ Heel erg

**191. Lichaamsgeur**<br><br>Hoe ERG was uw LICHAAMSGEUR op het SLECHTSTE MOMENT in de afgelopen 7 dagen?
❒ Helemaal niet ❒ Een beetje ❒ Matig ❒ Erg ❒ Heel erg

**Andere symptomen**<br><br>Hebt u andere symptomen die u wilt melden?
❒ Ja ❒ Nee → Einde van de vragenlijst

**Indien 'Ja', maak een lijstje van alle andere symptomen:**
**1.**
Hoe ERG was dit symptoom op het SLECHTSTE MOMENT in de afgelopen 7 dagen?<br>❒ Helemaal niet ❒ Een beetje ❒ Matig ❒ Erg ❒ Heel erg

**2.**
Hoe ERG was dit symptoom op het SLECHTSTE MOMENT in de afgelopen 7 dagen?<br>❒ Helemaal niet ❒ Een beetje ❒ Matig ❒ Erg ❒ Heel erg

**3.**
Hoe ERG was dit symptoom op het SLECHTSTE MOMENT in de afgelopen 7 dagen?<br>❒ Helemaal niet ❒ Een beetje ❒ Matig ❒ Erg ❒ Heel erg

**4.**
Hoe ERG was dit symptoom op het SLECHTSTE MOMENT in de afgelopen 7 dagen?<br>❒ Helemaal niet ❒ Een beetje ❒ Matig ❒ Erg ❒ Heel erg

**5.**
Hoe ERG was dit symptoom op het SLECHTSTE MOMENT in de afgelopen 7 dagen?<br>❒ Helemaal niet ❒ Een beetje ❒ Matig ❒ Erg ❒ Heel erg

Hartelijk dank voor uw medewerking aan dit onderzoek.

Controleer alstublieft of u geen vragen hebt overgeslagen. U kunt de vragenlijst versturen in de bijgevoegde antwoordenvelop. Mocht deze onverhoopt ontbreken dan kunt u de vragenlijst (zónder postzegel) versturen naar:

Catharina ziekenhuis
Afdeling Gynaecologie en Verloskunde t.a.v. Cynthia Hendrikse
Michelangelolaan 2
5623 EJ Eindhoven

Mocht u nog vragen hebben over de vragenlijst of andere vragen over dit onderzoek, dan kunt u contact opnemen met de studiecoördinator dr. Jurgen Piek, te bereiken op 040 - 239 93 00 of per email via stapover@catharinaziekenhuis.nl.
